# Quality of life under extended continuous versus intermittent adjuvant letrozole in lymph node-positive, early breast cancer patients: the SOLE randomised phase 3 trial

**DOI:** 10.1038/s41416-019-0435-4

**Published:** 2019-04-10

**Authors:** Karin Ribi, Weixiu Luo, Marco Colleoni, Per Karlsson, Jacquie Chirgwin, Stefan Aebi, Guy Jerusalem, Patrick Neven, Vincenzo Di Lauro, Henry L. Gomez, Thomas Ruhstaller, Ehtesham Abdi, Laura Biganzoli, Bettina Müller, Annelore Barbeaux, Marie-Pascale Graas, Manuela Rabaglio, Prudence A. Francis, Theodoros Foukakis, Olivia Pagani, Claudio Graiff, Daniel Vorobiof, Rudolf Maibach, Angelo Di Leo, Richard D. Gelber, Aron Goldhirsch, Alan S. Coates, Meredith M. Regan, Jürg Bernhard, on behalf of the SOLE Investigators

**Affiliations:** 1 Quality of Life Office, International Breast Cancer Study Group Coordinating Center, Bern, Switzerland; 2 International Breast Cancer Study Group Statistical Center, Department of Biostatistics and Computational Biology, Dana-Farber Cancer Institute, Boston, MA USA; 3 Division of Medical Senology, IEO, European Institute of Oncology IRCCS, Milan, Italy; 4 Department of Oncology, Institute of Clinical Sciences, Sahlgrenska Academy/Sahlgrenska University Hospital, University of Gothenburg, Gothenburg, Sweden; 5 Box Hill and Maroondah Hospitals, Monash University, Victoria, Australia; 6 Luzerner Kantonsspital, Lucerne, Switzerland; 7 CHU Liège, Liège University, Liège, Belgium; 8 Multidisciplinary Breast Center, University Hospitals, KU Leuven, Leuven, Belgium; 9 Division of Medical Oncology B, CRO-Aviano, Aviano, Italy; 10 Instituto Nacional de Enfermedades Neoplásicas, Lima, Peru; 11 Breast Center St. Gallen, Swiss Group for Clinical Cancer Research and International Breast Cancer Study Group, Bern, Switzerland; 12 The Tweed Hospital, Tweed Heads, NSW & Griffith University Gold Coast, Southport, Australia; 13 Hospital of Prato-AUSL Toscana Centro, Istituto Toscano Tumori, Prato, Italy; 14 Chilean Cooperative Group for Oncologic Research (GOCCHI), Providencia, Santiago, Chile; 15 CHR Verviers, Verviers, Belgium; 16 CHC Clinique St. Joseph, Liège, Belgium; 17 Bern University Hospital, Inselspital, Bern, Switzerland; 18 Peter MacCallum Cancer Center, University of Melbourne, Melbourne and Breast Cancer Trials Australia & New Zealand, University of Newcastle, Newcastle, Australia; 19 Department of Oncology, Karolinska Institute and University Hospital, Stockholm, Sweden; 20 Institute of Oncology of Southern Switzerland, Bellinzona, Geneva University Hospitals, Geneva, Swiss Group for Clinical Cancer Research (SAKK) and International Breast Cancer Study Group, Bern, Switzerland; 21 Division of Medical Oncology, Ospedale Centrale di Bolzano, Bolzano, Italy; 22 Sandton Oncology Centre, Johannesburg, South Africa; 23 International Breast Cancer Study Group Coordinating Center, Bern, Switzerland; 24 International Breast Cancer Study Group Statistical Center, Department of Biostatistics and Computational Biology, Dana-Farber Cancer Institute, Harvard Medical School, Harvard T.H. Chan School of Public Health and Frontier Science and Technology Research Foundation, Boston, MA USA; 25 International Breast Cancer Study Group and IEO, European Institute of Oncology IRCCS, Milan, Italy; 26 International Breast Cancer Study Group and University of Sydney, Sydney, Australia; 27 International Breast Cancer Study Group Statistical Center, Department of Biostatistics and Computational Biology, Dana-Farber Cancer Institute, Harvard Medical School, Boston, MA USA; 28 Quality of Life Office, International Breast Cancer Study Group Coordinating Center and Bern University Hospital, Inselspital, Bern, Switzerland

**Keywords:** Breast cancer, Human behaviour

## Abstract

**Background:**

In the phase III SOLE trial, the extended use of intermittent versus continuous letrozole for 5 years did not improve disease-free survival in postmenopausal women with hormone receptor-positive breast cancer. Intermittent therapy with 3-month breaks may be beneficial for patients’ quality of life (QoL).

**Methods:**

In the SOLE QoL sub-study, 956 patients completed the Breast Cancer Prevention Trial (BCPT) symptom and further QoL scales up to 24 months after randomisation. Differences in change of QoL from baseline between the two administration schedules were tested at 12 and 24 months using repeated measures mixed-models. The primary outcome was change in hot flushes at 12 months.

**Results:**

There was no difference in hot flushes at 12 months between the two schedules, but patients receiving intermittent letrozole reported significantly more improvement at 24 months. They also indicated less worsening in vaginal problems, musculoskeletal pain, sleep disturbance, physical well-being and mood at 12 months. Overall, 25–30% of patients reported a clinically relevant worsening in key symptoms and global QoL.

**Conclusion:**

Less symptom worsening was observed during the first year of extended treatment with the intermittent administration. For women experiencing an increased symptom burden of extended adjuvant endocrine therapy, an intermittent administration is a safe alternative.

**Clinical trial information:**

Clinical trial information: NCT00651456.

## Background

In animal models of breast cancer, acquired resistance to letrozole can be reversed by discontinuing treatment, suggesting a strategy of alternating “on-off” letrozole treatment as a therapeutic option to prolong sensitivity to endocrine therapy.^1^ The Study of Letrozole Extension (SOLE) study compared intermittent (3-month treatment breaks during the course in 4 out of 5 years) with continuous use of letrozole as extended adjuvant therapy in postmenopausal women with node-positive, hormone-receptor positive early breast cancer who remained free of relapse after 4–6 years of adjuvant endocrine therapy.

The recently published efficacy results showed no superiority in disease-free survival for the use of intermittent letrozole compared with the standard continuous administration.^2^ Previous trials showed that patients suffer from symptoms related to endocrine therapy during the standard treatment duration of five years in postmenopausal women with early breast cancer.^3,4^ An extension of endocrine treatment implies continuation of symptom burden with a potential physical and psychological impact on patients’ quality of life (QoL).

In the MA.17 extended-adjuvant trial,^5^ postmenopausal women who were randomised to five years of letrozole or placebo after five years of tamoxifen reported a worsening in mean change scores for a number of QoL domains.^6^ Small, but statistically significant differences between the letrozole and placebo group were seen for physical function, bodily pain, vitality, and sexuality up to two years on extended letrozole. Vasomotor symptoms improved over time for both groups but this seemed to be delayed for patients treated with letrozole.^6^ A minority of patients receiving either letrozole or placebo indicated symptoms to be bothersome.

Although some symptoms related to endocrine therapy persist, the majority of symptoms improve after treatment completion.^4^ Whether a therapy break is similarly beneficial for patients’ QoL is an open question in this setting. The aim of the SOLE QoL sub-study was to compare symptom-specific and global QoL in a subsample of patients randomly assigned to continuous or intermittent letrozole administration schedules and to compare the proportion of patients on each regimen who suffer from clinically-relevant symptom burden.

## Methods

### Study design and participants

SOLE is a multicentre, open-label, randomised phase 3 trial in 240 centres of the Breast International Group-affiliated cooperative groups in 22 countries.^2^ The prospectively-defined QoL sub-study was conducted in 60 International Breast Cancer Study Group (IBCSG) affiliated centres in 9 countries (Table S1). Eligible women had to be postmenopausal, clinically free of breast cancer at enrolment without evidence of recurrent disease at any time before randomisation. They had to have completed 4–6 years of previous adjuvant endocrine therapy within the past year with aromatase inhibitors (AI), selective oestrogen receptor modulators (SERMs), or a sequential combination of both. Detailed SOLE eligibility criteria are described elsewhere.^2^ Ethics committees and appropriate national health authorities from each centre approved the protocol, including this sub-study, and all patients provided written informed consent as part of the informed consent for the main trial.

### Procedures

Eligible women were randomly assigned 1:1 to receive continuous (2.5 mg/day orally for 5 years) or intermittent letrozole (2.5 mg/day orally for 9 months followed by a 3-month break in years 1–4 and then 2.5 mg/day during all 12 months of year 5). The QoL sub-study was activated at the start of the parent trial on November 8, 2007. The target QoL enrolment goal was met as of November 30, 2010, and the sub-study enrolment was closed as of December 31, 2010. However, sub-study enrolment continued until July 26, 2012 at centres enroling patients into a second SOLE sub-study, investigating changes in oestrogen levels and grip strength (NCT01281137). Because that sub-study had 1:3 enrolment of patients assigned to continuous versus intermittent letrozole use, the total enrolment in the QoL sub-study included a greater number of patients assigned to intermittent letrozole use. QoL assessments were mandatory for all patients included in SOLE at centres participating in the sub-study. Treatment compliance was assessed by case report forms, which collected the dates of beginning and end of all letrozole interruptions that lasted more than one month in duration. The monitoring of adherence was done according to the local standards of care.

### QoL assessment

Symptom-specific QoL was assessed by the Breast Cancer Prevention Trial (BCPT) symptom scales.^7^ They represent a refinement of the 42-item BCPT checklist^8^ reducing the original scale to eight symptom dimensions corresponding to the physical symptom dimensions associated with breast cancer treatment, chemoprevention, menopause or normal aging: hot flushes (two items), nausea (two items), bladder control (two items), vaginal problems (two items), musculoskeletal pain (three items), cognitive problems (three items), weight problems (two items), and arm problems (two items).^7^ Different versions of this scale have shown adequate psychometric properties,^7,9,10^ and have been used in large clinical trials.^8,11^ Patients had to indicate how much they were bothered by each of the 18 symptoms during the past 4 weeks on a 5-point severity scale (0 = not at all; 1 = slightly; 2 = moderately; 3 = quite a bit; 4 = extremely). The symptom dimensions are obtained as the mean of the corresponding two or three items.

The BCPT scales were complemented with further symptom-specific QoL scales related to endocrine treatment used in previous IBCSG trials, including tiredness, sleep disturbance, loss of sexual interest and difficulties becoming aroused.^12–14^ Global QoL was assessed with scales for physical well-being,^15,16^ mood,^15–17^ coping effort,^15,16,18^ and overall treatment burden.^19^ These additional scales were single-items in the linear analogue self-assessment (LASA) format.^16^ The clinical relevance of global LASA indicators has been confirmed in breast cancer trials in the adjuvant setting examining adjuvant standard chemotherapy,^20^ and endocrine therapy.^13,14,21^ As a reference measure for the BCPT hot flushes scale, we included the hot flushes LASA scale used in previous trials.^12–14,21^

For comparative purposes, we transformed all scales so that each score ranged from 0 to 100, with higher numbers reflecting a better condition. For interpretative purposes, hypotheses-related absolute BCPT scores are also presented in the original scale format. For the BCPT and IBCGS LASA scales, no established anchor-based cut-offs for a minimally-important difference (MID), defined as the smallest change in a QoL score considered important to patients, is available. Therefore, MIDs were calculated by using a one-half standard deviation of each scale in either direction based on the data of this study cohort, i.e., based on all observations of change from baseline to each time point (Table S2 shows MID of each BCPT symptom and LASA QoL scale).^22^

Patients completed the QoL forms at the clinic, before diagnostic procedures (exception: baseline) or treatment was given and regardless of disease status, at baseline and every six months up to two years after randomisation. Sub-study eligibility exceptions were cognitive or physical impairment that interfered with the QoL assessment, or inability to read any of the languages available on the forms.

### Prospectively-defined hypotheses

#### Primary

Twelve months after randomisation (i.e., after a 3-month break and before patients in the intermittent group start letrozole again) hot flushes will be worse in patients receiving continuous compared with those receiving intermittent letrozole.

#### Secondary

1. Twelve months after randomisation, both musculoskeletal and vaginal problems will be worse in patients receiving continuous compared with those receiving intermittent letrozole.
2. Starting and stopping treatment repeatedly over 2 years for patients randomised to receive intermittent letrozole is more burdensome on a global QoL level compared with patients receiving continuous letrozole.

### Statistical analysis

Analyses of the QoL sub-study were performed by the intention-to-treat principle. QoL forms were expected regardless of disease status, unless the patient died, withdrew consent for all trial participation, or was lost to follow-up.

The sample size was prospectively estimated on the basis of a small between-group difference of the change from baseline to 12 months in the hot flushes scale, estimating that 676 patients were needed to achieve 90% power to detect an effect size of 0.25 between the two groups on the basis of a two-sided 0.05 level *t*-test. To allow for a 10% non-compliance rate for QoL form submission, the target enrolment was inflated to 744 patients.

For each QoL scale, the change from baseline to each timepoint was calculated as timepoint minus baseline score, with negative changes representing a deterioration in scores. The changes were summarised as the mean with 95% CI at each timepoint, with focus on the change from baseline to 12 and 24 months.

Mixed-effects linear regression modelling for repeated measures was used to test the effect of treatment on changes in each of the QoL scales. All available data were analysed without imputation of missing data. Covariance structures including unstructured, AR(1) and Toeplitz were considered for the repeated measures over these assessment time points. An unstructured covariance structure was used to account for correlation of repeated measures. The model included treatment assignment (intermittent versus continuous use of letrozole), assessment time point (categorical: 6, 12, 18, 24 months), and the interactions of the two variables. Models were adjusted for patient and disease characteristics: age and body-mass index at randomisation, tumour size, tumour grade, ER/PgR status subgroup, HER2 status, number of positive lymph nodes, type and duration of prior endocrine therapy, and interval of time since the cessation of prior endocrine therapy until randomisation. From the model, an estimated mean difference between treatment groups was calculated with 95% CI for each time point and compared at 12 and 24-month using model contrasts. The reported *P* values were not adjusted for testing of multiple QoL scales over time to look for consistency of the signal among comparable QoL scales.

A prospectively-defined response analysis aimed at identifying the proportion of patients indicating a MID. Patients were categorised according to improved, no change, or worsened scores at 12 and 24 months post-randomisation, separately for each treatment group.

The effect of other covariates on changes of QoL from baseline was explored by looking at the consistency of pattern of the same covariate, using the significance level of 0.05, not adjusting for multiple comparisons. Further exploratory analysis addressed the correlation and responsiveness between the BCPT hot flushes and the corresponding LASA scale.

## Results

### Patient and disease characteristics

**Fig. 1:**
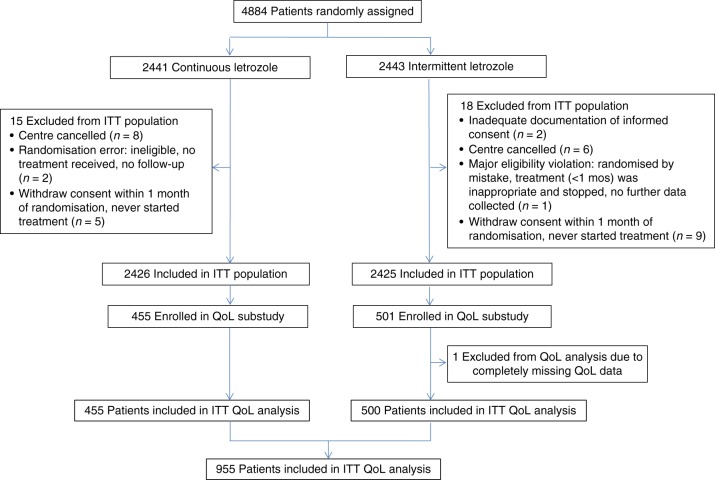
CONSORT flowchart to identify the 955-patient intention-to-treat (ITT) quality of life population. QoL, quality of life

Of the 4851 patients in the intention-to-treat SOLE trial population, 956 were enroled in the QoL sub-study between December 2007 and July 2012. After exclusion of one patient who had QoL data completely missing, 955 patients were included in the analysis (455 from the continuous, and 500 from the intermittent letrozole group; Fig. 1).

Patients who participated in the QoL sub-study tended to be younger than those who did not (median age 58 years [IQR 53–66] vs. 60 years [54–67]), were more often premenopausal at diagnosis, (27 vs. 22%), and more likely to have received previous SERMs only rather than AIs only or both (27 vs. 16%). The distributions of disease characteristics and the estimated treatment effect on disease-free survival were similar between those who participated and those who did not (data not shown). At the time of analysis, the median follow-up of the SOLE trial was 60 months (IQR, 53–72).^2^

Patient and disease characteristics at randomisation were balanced between treatment groups (Table 1). Before randomisation 27% of patients had received an AI, 27% SERMs only, and 46% both. The median duration of previous adjuvant endocrine therapy was 5 years in the continuous (IQR, 4.8–5.2) and in the intermittent group (IQR, 4.9−5.2). The median duration from the end of prior endocrine therapy to randomisation was 0.2 months in the continuous (IQR, 0.0–2.0) and 0.3 months in the intermittent group (IQR, 0.0−1.8). The QoL submission rate was 92% across all timepoints and similar between treatment groups (93% in the continuous vs. 91% in the intermittent group, Table S3).

**Table 1:** Patient and disease characteristics of patients in the SOLE quality of life substudy intention-to-treat (ITT) population, overall and according to treatment assignment

	Continuous letrozole (*n* = 455)		Intermittent letrozole (*n* = 500)		Overall (*n* = 955)	
*Age at randomisation (years)*						
<55	143	(31%)	163	(33%)	306	(32%)
55–59	114	(25%)	99	(20%)	213	(22%)
60–64	73	(16%)	96	(19%)	169	(18%)
65–69	62	(13%)	74	(15%)	136	(14%)
70+	63	(13%)	68	(14%)	131	(14%)
*Body mass index at randomisation*						
Normal (<25)	173	(38%)	214	(43%)	387	(41%)
Overweight (25 to <30)	164	(36%)	160	(32%)	324	(34%)
Obese (≥30)	95	(21%)	105	(21%)	200	(21%)
Unknown	23	(5%)	21	(4%)	44	(5%)
*Number of positive lymph nodes*						
0	1	(<1%)	3	(<1%)	4	(<1%)
1–3	307	(68%)	340	(68%)	647	(68%)
4–9	104	(23%)	110	(22%)	214	(22%)
≥10	43	(10%)	47	(9%)	90	(9%)
Unknown	1	(<1%)	3	(<1%)	4	(<1%)
*Tumour grade*						
1	80	(18%)	102	(20%)	182	(19%)
2	242	(53%)	254	(51%)	496	(52%)
3	119	(26%)	126	(25%)	245	(26%)
Unknown	14	(3%)	18	(4%)	32	(3%)
*Tumour size*						
≤2 cm	207	(46%)	237	(47%)	444	(47%)
>2 cm	244	(54%)	260	(52%)	504	(53%)
Unknown	4	(<1%)	3	(<1%)	7	(<1%)
*HER2 status*						
Negative	328	(72%)	366	(73%)	694	(73%)
Positive	75	(17%)	67	(13%)	142	(15%)
Unknown or not done	52	(11%)	67	(13%)	119	(13%)
*Prior chemotherapy*						
No	70	(15%)	77	(15%)	147	(15%)
Yes	385	(85%)	423	(85%)	808	(85%)
*Prior endocrine therapy*						
SERM(s) only	122	(27%)	136	(27%)	258	(27%)
Both SERM(s) and AI(s)	215	(47%)	225	(45%)	440	(46%)
AI(s) only	118	(26%)	139	(28%)	257	(27%)
*Duration of prior endocrine therapy*						
<4.5 years	56	(12%)	56	(11%)	112	(12%)
4.5–5.5 years	354	(78%)	393	(79%)	747	(79%)
>5.5 years	44	(10%)	51	(10%)	95	(10%)
Unknown	1	(<1%)	–	–	1	(<1%)
*Time from end of prior endocrine therapy to randomisation*						
≤1 month	296	(65%)	330	(66%)	626	(66%)
>1 month	159	(35%)	170	(34%)	329	(35%)

*ER* oestrogen receptor, *PgR* progesterone receptor, *HER2* human epidermal growth factor receptor 2, *AI* aromatase inhibitor, *SERM* selective oestrogen receptor modulator

In the QoL population, the reasons for early discontinuation of AI were indicated as adverse events or side-effects for 161 (17%) of 955 patients (86 [17%] of 500 in the intermittent letrozole group vs. 75 [16%] of 455 in the continuous letrozole group) and as patient's decision for 58 (7%) patients (28 [6%] vs. 30 [7%]).

### Changes in symptom-specific and global QoL over time

**Fig. 2:**
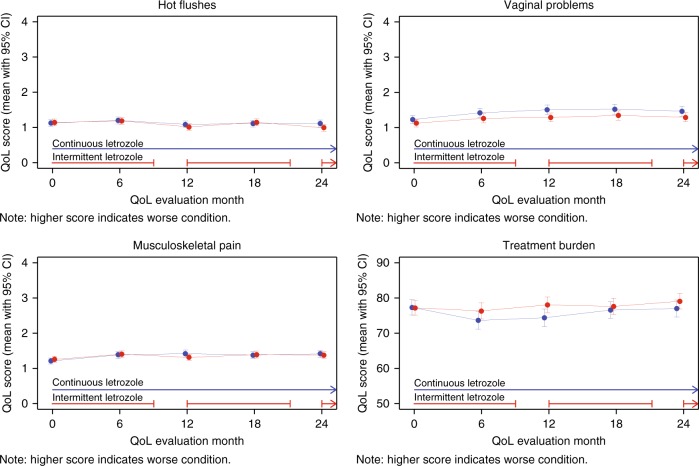
Absolute scores (mean with 95% CI) from baseline to 24 months according to treatment assignment for BCPT symptom scales of hot flushes, vaginal problems, and musculoskeletal pain (score range 0–4; higher scores indicating a worse condition), and QoL LASA indicator for treatment burden (score range 0–100; higher score indicates better condition. CI, confidence interval; QoL, quality of life; BCPT, Breast Cancer Prevention Trial; LASA, linear analogue self-assessment

Baseline QoL scores were similar between treatment groups (Table 2). On average women reported scores between 0 and 1 for most symptom scales at baseline, which would correspond to being bothered “not at all” to “slightly”. For specific symptom scales (hot flushes, vaginal problems and musculoskeletal pain), mean scores at baseline were between 1 and 2, which would correspond to being bothered “slightly” to “moderately”. Absolute QoL changes over the two-year observation period were marginal for BCPT hot flushes, vaginal problems, musculoskeletal pain scales, and LASA treatment burden (Fig. 2). For most of the other scales patients reported rather stable scores in both groups (not shown).

**Table 2:** Quality of life scales at baseline according to treatment assignment

Quality of life scales	Continuous letrozole (*n* = 455)		Intermittent letrozole (*n* = 500)		Continuous letrozole (*n* = 455)		Intermittent letrozole (*n* = 500)	
	Original score range^a^				Transformed score range^b^			
	Mean	SD	Mean	SD	Mean	SD	Mean	SD
*BCPT symptom scales* ^a^								
Hot flushes	1.1	1.0	1.1	1.1	71.8	24.6	71.5	27.0
Nausea	0.1	0.3	0.1	0.4	97.0	8.5	96.4	10.2
Bladder control	0.5	0.8	0.5	0.7	86.8	19.0	86.5	18.1
Vaginal problems	1.2	1.2	1.1	1.2	69.2	31.1	71.8	30.4
Musculoskeletal pain	1.2	0.9	1.3	1.0	69.8	23.0	68.6	24.0
Cognitive problems	1.0	0.9	1.0	0.9	74.3	21.6	75.1	22.5
Weight problems	1.0	1.0	0.9	1.0	74.7	24.6	76.8	24.7
Arm problems	0.6	0.8	0.6	0.8	85.6	19.5	85.1	19.9
*LASA symptom scales*								
Hot flushes					69.1	28.4	68.6	30.4
Sleep disturbance	–	–	–	–	68.0	26.6	67.7	28.9
Tiredness	–	–	–	–	62.6	27.6	63.7	26.4
Difficulties in becoming aroused	–	–	–	–	64.2	30.1	66.7	29.7
Loss of sexual interest	–	–	–	–	54.3	33.4	55.9	34.2
*LASA global scales*								
Physical well-being	–	–	–	–	78.2	20.7	77.6	22.2
Mood	–	–	–	–	78.0	22.0	76.8	23.2
Coping effort	–	–	–	–	79.0	23.6	76.8	81.3
Treatment burden	–	–	–	–	77.5	23.5	77.4	23.4

*SD* standard deviation

^a^BCPT original scales (i.e., symptom dimensions obtained by the mean of the corresponding two or three items) range from 0 to 4

^b^Transformed BCPT and LASA scales range from 0 to 100, with higher scores indicating a better condition

### Treatment comparisons

**Fig. 3:**
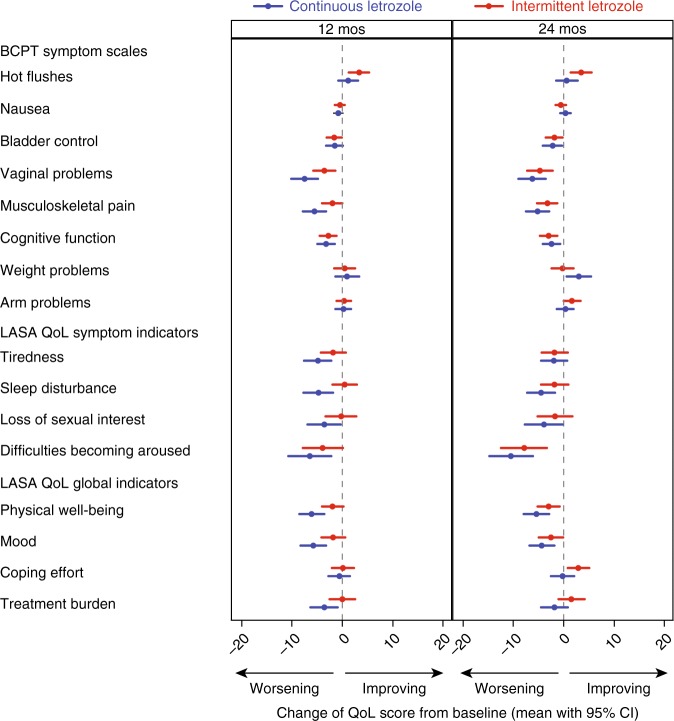
Change of scores in BCPT symptom scales and symptom-specific and global QoL LASA scales from baseline to 12 and 24 months (mean with 95% CI), according to treatment assignment (BCPT symptom scales were recalculated to 0-100 range before calculating the change). CI, confidence interval; QoL, quality of life; BCPT, Breast Cancer Prevention Trial; LASA, linear analogue self-assessment

Treatment comparison in changes from baseline are presented in Fig. 3, Tables S4 and S5. BCPT symptom scales were recalculated to 0-100 range before calculating the change. Regarding our primary hypothesis, we found no differences in mean changes from baseline for BCPT hot flushes between treatment groups at 12 months (mean ∆:2, 95% CI [−1 to +5]; *P* = 0.11). At month 24 (i.e., after the second interruption) patients assigned intermittent letrozole reported a greater improvement in hot flushes (3, 95% CI [0–6]; *P* = 0.025) than those assigned continuous letrozole. The two measures for flushes (BCPT scale and IBCSG LASA) showed similar responsiveness to changes over time and treatment effects, respectively (Table S7; Figure S1).

At 12 months, patients receiving intermittent letrozole reported significantly less worsening in BCPT vaginal problems (mean ∆: 4 [95% CI 1–8]; *P* = 0.017), and musculoskeletal pain (3 [0–6]; *P* = 0.023) compared to patients in the continuous group (secondary hypothesis 1). No differences between groups were seen for LASA treatment burden at month 12 (3, 95% CI [0–7]; *P* = 0.082) or month 24 (3 [0–7]; *P* = 0.082; secondary hypothesis 2). The intermittent group also reported significantly less worsening for LASA sleep disturbance (5 [95% CI 1–9]; *P* = 0.007), physical well-being (4 [1–8]; *P* = 0.008) and mood (4 [0–7]; *P* = 0.026). For all other scales, no significant differences in  changes were seen between groups at any of the timepoints.

### Response analysis

**Fig. 4:**
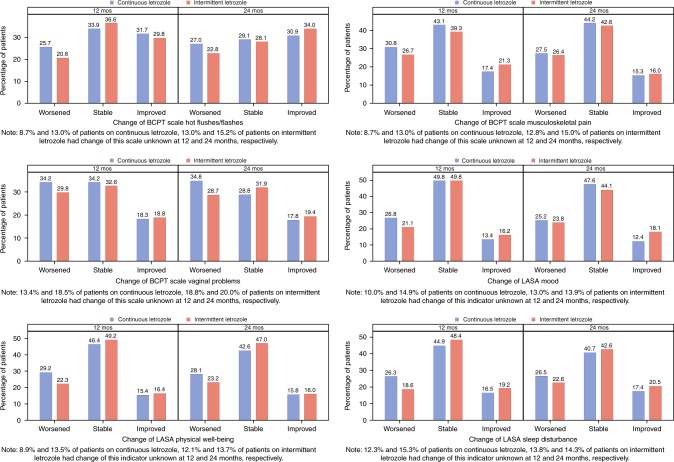
Proportion of patients who reported clinically-relevant worsened, stable or improved scores for selected quality of life scales at 12 and 24 months according to treatment assignment

The clinical relevance of these results is shown by a response analysis for those endpoints with significant treatment differences (Fig. 4; Table S6). For BCPT vaginal problems on average one-third of patients reported a clinically relevant worsening at 12 and 24 months. A smaller proportion of patients reported a worsening for BCPT musculoskeletal pain and LASA sleep disturbances at both time points. A minority of patients reported improved scores for these symptoms. In contrast, around one-third of the patients reported improved scores for BCPT hot flushes at 12 and 24 months. On average one-fourth of patients reported a worsening in LASA physical well-being and mood at 12 and 24 months. Overall, proportions of patients reporting a worsening in QoL were higher in the continuous than in the intermittent group.

### Predictors for symptom-specific and global QoL

Among the patient and disease characteristics, only a few showed a noteworthy impact on changes in QoL scores. Type of prior endocrine therapy (AI alone, SERM alone, both SERM and AI) significantly predicted changes in musculoskeletal pain (*P* < 0.0001), vaginal problems (*P* < 0.0001), loss of sexual interest (*P* < 0.0007) and overall symptom burden (*P* = 0.026), with patients who received prior SERM(s) only reporting a greater worsening in these scales compared to patients who received prior AI(s) only. Patients who were randomised within one month compared to those randomised more than one month from the end of prior endocrine therapy had significantly less worsening of bladder control (*P* = 0.041), hot flushes (*P* = 0.033), coping effort (*P* = 0.050), and tiredness (*P* = 0.008). BMI predicted weight problems (*P* = 0.012) indicating that patients who were overweight or obese had less worsening compared with patients having normal BMI. We found no effect of age on any of the QoL scales, except for the BCPT nausea scale (*P* = 0.004). Patients who were between 55 and 59 years old reported a greater worsening of nausea compared to patients who were younger than 55 years.

## Discussion

In general, our results show less worsening of symptom-specific and global QoL in patients assigned to intermittent letrozole compared with those assigned to continuous letrozole. Differences in hot flushes, vaginal problems, musculoskeletal pain, sleep disturbances, physical well-being and mood were a result of small improvements in the intermittent group during the first or the second letrozole break, respectively. Changes over time and treatment differences did not reach clinical relevance. Our findings are in line with those of MA.17 showing statistically significant but small treatment differences in favour of the placebo group for physical functioning at 12 months, bodily pain and vitality at 6 months, vasomotor symptoms up to 24 months, and sexual problems at 12 and 24 months.^6^

In SOLE, after completing 4–6 years of prior adjuvant endocrine therapy, two-thirds of patients had a treatment-free period of less than one month. The impact of treatment on symptom-specific QoL after restarting endocrine therapy is expected to be less substantial than in a first-time administration. The baseline scores for some symptoms (hot flushes, vaginal problems, musculoskeletal pain, cognitive problems, weight gain assessed by the BCPT scale) were worse than those of reference data from a sample of 208 women at risk but without a diagnosis for breast cancer,^7^ suggesting that patients started extended therapy with some severity of key symptoms. A deterioration on this level, even if small, can be detrimental to QoL. Our QoL response analysis showed, regardless of treatment, that approximately 25 to 30% of patients indicated a substantial worsening depending on symptom or QoL scale. In MA.17, 29% of patients on extended letrozole reported a clinically-relevant worsening for vasomotor symptoms vs. 22% on placebo on at least one occasion during the five years on letrozole.^6^ Corresponding proportions of a clinically-relevant worsening were 43% (for letrozole) vs. 38% (for placebo) for the physical domain, 39 vs. 37% for the psychosocial domain, and 32 vs. 29% the sexual domain.^6^ Differences in the proportions of patients who experienced a relevant worsening in QoL between extended letrozole and placebo were between 2 and 7% depending on QoL scale. In SOLE, we observed slightly higher differences in these proportions between the intermittent and continuous letrozole groups (4–8% depending on scale), with all favouring the intermittent group. The proportions of patients with a clinically-relevant symptom burden in the MA.17 are not directly comparable to our results due to different QoL instruments used.

Comparing the magnitude of QoL differences between extended intermittent and continuous letrozole to the findings of randomised trials investigating the QoL effects of the standard 5 year administration of an AI vs. tamoxifen is complicated.^3,4,11,23^ These trials lacked the discussion of the clinical relevance of the treatment-related differences and used varying instruments to assess patient-reported symptoms. In NSABP B-35, using the BCPT symptom checklist, 15–20% of patient with ductal carcinoma in situ reported hot flushes, 15% vaginal dryness, 32% joint pain, 28%, muscle stiffness to be “quite a bit” or “very much” bothersome after 6 months on anastrozole treatment.^11^ Independent of the QoL measure used or how individual symptom severity is reported, evidence suggests that an important subgroup is considerably affected by symptom burden.

The SOLE trial has shown that recurring short therapy breaks have no benefit for patients regarding disease outcomes.^2^ According to the international consensus guidelines for breast cancer in postmenopausal women, the use of an AI for 10 years should be discussed on an individual basis.^24^ Our QoL results support this recommendation by stressing the importance of an individually-adjusted therapy for those patients who suffer the most. We did not find any indication that starting and stopping treatment is more burdensome on a global QoL level (i.e., overall treatment burden) compared to the continuous administration. This is also reflected in the adherence rates reported for the overall SOLE population: 98% of patients who interrupted letrozole after nine months resumed treatment according to protocol after the 3 months gap in year 1, 97% in year 2, respectively.^2^

Our analysis of potential predictors of QoL changes in the extended setting revealed that women who had SERM(s) only as prior endocrine therapy or who were randomised more than one month after completing prior endocrine therapy experienced a greater worsening in selected QoL scales. Changing the type of or re-initiating endocrine treatment after a longer break may cause hormonal changes that aggravate the symptom experience again.

SOLE is the first trial comparing patient-reported symptoms and global QoL between continuous and intermittent extended endocrine therapy. Strengths are the prospectively-defined hypotheses and the high QoL forms submission rates. As an inevitable result of the trial design, there was no placebo control group. Our results indicate that the difference in proportion of patients with a clinically important worsening in symptom burden between intermittent and continuous letrozole is comparable to those observed between continuous letrozole and placebo in MA.17. We assessed QoL only during the first two years of extended treatment covering two treatment breaks. Significant effects were observed only after the first interruption, except for hot flushes. Whether these effects may occur again after the third or fourth break is not known. In MA.17, significant differences in QoL between extended letrozole and placebo were observed only during the first two of five years.^6^ In the MA.17R trial, treatment with letrozole extended to 10 years compared to placebo did not show significant differences in overall QoL, most of the QoL domains and menopause-related health.^23,25^

Due to the lack of an established criterion defining clinically-relevant changes of the BCPT symptom scales, we used the standard measure of one-half of a standard deviation, which we consider a rather conservative estimate, underestimating the proportion of patients suffering from specific endocrine symptoms. The small positive change of hot flushes at 24 months and the modest difference between the randomised treatments were confirmed by the reference measure for hot flushes (LASA scale). Future trials should include methodological questions in order to investigate the clinical relevance of smaller, and in particular negative changes.^26^

In conclusion, changes in symptom-specific and global QoL were small. For several symptoms and global QoL scales, significantly less worsening was observed with the intermittent administration of letrozole, mainly during the first year of extended treatment. This was confirmed by the higher proportion of patients in the continuous group who experienced a clinically-important worsening. Since the efficacy of the intermittent and continuous letrozole regimens did not differ, for women experiencing an increased endocrine symptom burden of extended adjuvant endocrine therapy, an intermittent administration is a safe alternative.

## Supplementary information

Supplementary figures and tables

## Data Availability

The data on which these analyses are based forms part of the clinical trials database of the International Breast Cancer Study Group, and as such is available for use on application in accordance with IBCSG data sharing policy.
