# Correction: Quality of life under extended continuous versus intermittent adjuvant letrozole in lymph node-positive, early breast cancer patients: the SOLE randomised phase 3 trial

**DOI:** 10.1038/s41416-019-0709-x

**Published:** 2020-01-16

**Authors:** Karin Ribi, Weixiu Luo, Marco Colleoni, Per Karlsson, Jacquie Chirgwin, Stefan Aebi, Guy Jerusalem, Patrick Neven, Vincenzo Di Lauro, Henry L. Gomez, Thomas Ruhstaller, Ehtesham Abdi, Laura Biganzoli, Bettina Müller, Annelore Barbeaux, Marie-Pascale Graas, Manuela Rabaglio, Prudence A. Francis, Theodoros Foukakis, Olivia Pagani, Claudio Graiff, Daniel Vorobiof, Rudolf Maibach, Angelo Di Leo, Richard D. Gelber, Aron Goldhirsch, Alan S. Coates, Meredith M. Regan, Jürg Bernhard, on behalf of the SOLE Investigators

**Affiliations:** 1 Quality of Life Office, International Breast Cancer Study Group Coordinating Center, Bern, Switzerland; 2 International Breast Cancer Study Group Statistical Center, Department of Biostatistics and Computational Biology, Dana-Farber Cancer Institute, Boston, MA USA; 3 Division of Medical Senology, IEO, European Institute of Oncology IRCCS, Milan, Italy; 4 Department of Oncology, Institute of Clinical Sciences, Sahlgrenska Academy/Sahlgrenska University Hospital, University of Gothenburg, Gothenburg, Sweden; 5 Box Hill and Maroondah Hospitals, Monash University, Victoria, Australia; 6 Luzerner Kantonsspital, Lucerne, Switzerland; 7 CHU Liège, Liège University, Liège, Belgium; 8 Multidisciplinary Breast Center, University Hospitals, KU Leuven, Leuven, Belgium; 9 Division of Medical Oncology B, CRO-Aviano, Aviano, Italy; 10 Instituto Nacional de Enfermedades Neoplásicas, Lima, Peru; 11 Breast Center St. Gallen, Swiss Group for Clinical Cancer Research and International Breast Cancer Study Group, Bern, Switzerland; 12 The Tweed Hospital, Tweed Heads, NSW & Griffith University Gold Coast, Southport, Australia; 13 Hospital of Prato-AUSL Toscana Centro, Istituto Toscano Tumori, Prato, Italy; 14 Chilean Cooperative Group for Oncologic Research (GOCCHI), Providencia, Santiago, Chile; 15 CHR Verviers, Verviers, Belgium; 16 CHC Clinique St. Joseph, Liège, Belgium; 17 Bern University Hospital, Inselspital, Bern, Switzerland; 18 Peter MacCallum Cancer Center, University of Melbourne, Melbourne and Breast Cancer Trials Australia & New Zealand, University of Newcastle, Newcastle, Australia; 19 Department of Oncology, Karolinska Institute and University Hospital, Stockholm, Sweden; 20 Institute of Oncology of Southern Switzerland, Bellinzona, Geneva University Hospitals, Geneva, Swiss Group for Clinical Cancer Research (SAKK) and International Breast Cancer Study Group, Bern, Switzerland; 21 Division of Medical Oncology, Ospedale Centrale di Bolzano, Bolzano, Italy; 22 Sandton Oncology Centre, Johannesburg, South Africa; 23 International Breast Cancer Study Group Coordinating Center, Bern, Switzerland; 24 International Breast Cancer Study Group Statistical Center, Department of Biostatistics and Computational Biology, Dana-Farber Cancer Institute, Harvard Medical School, Harvard T.H. Chan School of Public Health and Frontier Science and Technology Research Foundation, Boston, MA USA; 25 International Breast Cancer Study Group and IEO, European Institute of Oncology IRCCS, Milan, Italy; 26 International Breast Cancer Study Group and University of Sydney, Sydney, Australia; 27 International Breast Cancer Study Group Statistical Center, Department of Biostatistics and Computational Biology, Dana-Farber Cancer Institute, Harvard Medical School, Boston, MA USA; 28 Quality of Life Office, International Breast Cancer Study Group Coordinating Center and Bern University Hospital, Inselspital, Bern, Switzerland

**Keywords:** Breast cancer, Human behaviour

**Correction to:** *British Journal of Cancer* (2019) **120**, 959–967; 10.1038/s41416-019-0435-4; published online 10 April 2019.

The ClinicalTrials.gov Identifier was incorrectly listed as NCT00651456. The correct identifier is listed below.

**Clinical trial information**

Clinical trial information: NCT00553410.

