# Supplementary material for: Quality of life under extended continuous versus intermittent adjuvant letrozole in lymph node-positive, early breast cancer patients: the SOLE randomised phase 3 trial
Source: Br J Cancer. 2019 Apr 10;120(10):959–67. doi: 10.1038/s41416-019-0435-4 (PMC6734915; doi:10.1038/s41416-019-0435-4)
Supplement: Supplementary file 1 — Supplementary figures and tables [file 41416_2019_435_MOESM1_ESM.docx]

**Ribi et al.**

**Quality of life under extended continuous versus intermittent adjuvant letrozole in lymph node-positive, early breast cancer patients: the SOLE randomized phase 3 trial**

**ONLINE ONLY MATERIAL**

**Section 1**

**The** **International Breast Cancer Study Group (IBCSG) Participating Centers and Principal Investigators for the Quality-of-Life Substudy**

Ethics committees and appropriate national health authorities from each center approved the protocol, including the SOLE substudy. All participating centers are listed below. Patients provided written informed consent as part of the informed consent for the main trial.

**Belgium (Accrual 231)**

Cliniques Universitaires Saint-Luc, Brussels; M. Berliere

UZ Gasthuisberg, Leuven; P. Neven

CHU Sart-Tilman, Liège; G. Jerusalem

Clinique St. Elisabeth, Namur; P. Vuylsteke

Clinique Saint- Joseph, Liège; M.P. Graas

AZ Klina, Brasschaat; D. Verhoeven

Jules Bordet Institute, Bruxelles; A. Gombos

CHR Verviers, Verviers; A. Barbeaux

CHR Citadelle, Liège; J.P. Salmon

**Italy (Accrual 203)**

Centro di Riferimento Oncologico (CRO), Aviano; V. DiLauro

IEO, Istituto Europeo di Oncologia IRCCS, Milano; M. Colleoni

Ospedale Degli Infermi, Biella; M. Clerico

Istituti Clinici Scientifici Maugeri, Pavia; A. Bernardo

Ospedale Di Prato, Azienda USL Toscana Centro, Prato; L Biganzoli

E.O. Ospedali Galliera, Genova; A. Gennari

Azienda Sanitaria dell’Alto Adige-Sanitaetsbetrieb Suedtirol, Ospedale Centrale di Bolzano; C. Graiff

Istituto Scientifico Romagnolo per lo Studio e la Cura dei Tumori, Meldola; D. Amadori

**Breast Cancer Trials Australia & New Zealand (BCT-ANZ; Accrual 171),** J. Chirgwin, P. Francis, J. Forbes

**Australia (Accrual 164)**

Peter MacCallum Cancer Centre, Melbourne; P. Francis

Box Hill Hospital, Melbourne; J. Chirgwin

Maroondah Hospital, Melbourne; J. Chirgwin

Fiona Stanley Hospital (FSH), Murdoch; A. Redfern

Calvary Mater Newcastle Hospital, Newcastle; A. van der Westhuizen

Prince of Wales Hospital, Newcastle; C. Lewis

Concord Repatriation General Hospital, Sydney; P. Beale

Royal Hobart Hospital, Hobart; I. Byard

Port Macquarie Base Hospital, Port Macquarie; S. Begbie

Tamworth Rural Referral Hospital, North Tamworth; F. Sardelic

The Tweed Hospital, Tweed Heads; E. Abdi

The Breast & Endocrine Centre, Gateshead; D. Clark

North West Regional Hospital, Burnie; A. Chindewere

Southern Highlands Cancer Centre, Bowral; S. Della-Fiorentina

**New Zealand (Accrual 7)**

Waikato Hospital, Waikato; L. Gilbert

Christchurch Hospital, Christchurch; K. Gardner

**Switzerland (Accrual 113)**

Inselspital, Bern; M. Rabaglio

Thun-Berner Oberland, Thun; D. Rauch

Oncocare Dr. Buser, Bern; K. Buser

Stadtspital Triemli, Zürich; S. Von Orelli

Kantonsspital St. Gallen, St. Gallen; T. Ruhstaller

Tumor - und Brustzentrum ZeTup St. Gallen, St. Gallen; H.J. Senn

Oncology Institute of Southern Switzerland, Bellinzona; O. Pagani

Centre Hospitalier Universitaire Vaudois, Lausanne; K. Zaman

Kantonsspital Aarau; E. Kralidas

**Sweden (Accrual 106)**

Lidk*ö*ping Hospital, Lidköping; P. Nyman

Skaraborg Hospital Skövde, Skövde; A. Jungquist

Sahlgrenska University Hospital, Gothenburg; P. Karlsson

Southern Älvsborg Hospital, Borås; C. Chamalidou

Karolinska University Hospital, Solna; T. Foukakis

Malar Hospital, Eskilstuna; A. Valachis

**Grupo Oncológico Cooperativo Chileno de Investigación (GOCCHI; Accrual 68), Chile:** S. Torres Castro, B. Müller, J. Retamales, Z. Zlatar

Instituto Nacional del Cancer, Santiago; R. Torres Ulloa

Fundacion Arturo Lopez Perez, Santiago; M. Fritis

Instituto De Radiomedicina, Vitacura; S. Sole

Hospital San Juan de Dios, Santiago; S. Torres Castro

Hospital San Borja Arriaran, Santiago; J. Letzkus

Hospital Dr. Luis Tisne Brousse, Santiago; P.A. Escobar Oliva

Hospital Carlos Van Buren, Valparaíso; J. Arancibia

Hospital Base de Valdivia, Valdivia; J.B. Cardemil

**Peru (Accrual 39)**

Instituto de Enfermedades Neoplasicas, Lima; H. Gomez

**South Africa (Accrual 24)**

Cape Town, Cape Town; J. Wetter

Sandton Oncology Center, Johannesburg; D. Vorobiof

The Head, Neck And Breast Clinic, Panorama; J. Apffelstaedt

**International Breast Cancer Study Group**

**SOLE Steering Committee**: M Colleoni (Chair); S Aebi, J Chirgwin, P Karlsson (Co-Chairs); A. Mathieu-Boue (Novartis), M Bellet, L Blacher, A Coates, A Di Leo, R Gelber, M Gnant, A Goldhirsch, A Hiltbrunner, G Jerusalem, C Kamby, R Kammler, I Kössler, K Kuroi, S Loibl, W Luo, R Maibach, J Martinez, B Müller, P Neven, M Rabaglio, M Regan, B Ruepp, K Scott, H Shaw, E Simoncini, A Thompson, G Viale

**IBCSG** **Scientific Committee:** M Colleoni (Chair), A Di Leo (Co-Chair)

**IBCSG** **Scientific Executive Committee:** M Colleoni, A Di Leo, F Boyle, G Jerusalem, M Regan, G Viale

**IBCSG Foundation Council:** R Stahel (President), S Aebi, F Boyle, A Coates, M Colleoni, A Di Leo, R Gelber, A Goldhirsch, G Jerusalem, P Karlsson, I Kössler, I Láng, M Regan

**IBCSG Coordinating Center, Bern, Switzerland:** A Hiltbrunner (Director), Y Chittazhathu Kurian Kuruvilla, I Driesang, L Fritzsche, A Gasca, R Kammler, R Maibach, R Pfister, M Rabaglio, S Ribeli-Hofmann, B Ruepp, M Weber

**IBCSG Statistical Center, Department of Biostatistics and Computational Biology, Dana-Farber Cancer Institute, Boston, MA, USA**: M Regan (Director), J Aldridge, R Gelber, H Huang, W Luo, C Mahoney, K Price

**IBCSG Data Management Center, Frontier Science & Technology Research Foundation, Amherst, NY, USA:** L Blacher (Director), K Scott (DM Section Head), H Shaw (Lead TC), M Blackwell, M Caporale, M Greco, R Hecker, A Karausch, C King, S Lippert, J Meshulam, M Metz, K Rickard, T Scolese, R Starkweather, Y Veira, D Weinbaum, C Westby, T Zielinski

**IBCSG Quality of Life Office, Bern, Switzerland**: J Bernhard, K Ribi

**IBCSG Central Pathology Office, IEO, European Institute of Oncology IRCCS, Division of Pathology, Milan**: G Viale (Director), S Andrighetto, G Bardeli, F Ciocca, P Dell'Orto, L Russo

**SOLE fellows**: D Bretel (GECO-Peru), M Cinefra (Italy), S Gallant (SCTBG), J Håkansson (WSBCG, Sweden), D Lindsay (ANZBCTG), S. Loibl (GBG), C Mariani (GOCCHI), F Olivier (Belgium), L McMichael (ZA), E Nel (ZA), A Polo (SOLTI), A Raaberg (DBCG)

**Breast International Group (BIG):** M Piccart-Gebhart, J Martinez

**Section 2: Supplementary Tables and Figures**

**Table S1.** Numbers of enrolling centres and numbers of patients enrolled in the SOLE quality-of-life substudy, according to country

|  |  |  |
| --- | --- | --- |
| **Country** | **Number of Centres** | **Number of Patients** |
| Belgium | 9 | 231 |
| Italy | 8 | 203 |
| Australia | 14 | 164 |
| New Zealand | 2 | 7 |
| Switzerland | 9 | 113 |
| Sweden | 6 | 106 |
| Chile | 8 | 68 |
| Peru | 1 | 39 |
| South Africa | 3 | 24 |
| Overall | 60 | 955 |

**Note:** Centres in Austria, Denmark, France, Germany, Hungary, India, Ireland, Japan, Russia, Slovenia, Spain, United Kingdom, USA did not participate in the substudy.

**Table S2**: Minimally-important differences (MID) of each BCPT symptom and LASA QoL scale based on half standard deviation for change from baseline

| **Change from baseline** | **Min** | **Max** | **std** | **Half std** |
| --- | --- | --- | --- | --- |
| **BCPT Symptom Scales** |  |  |  |  |
| Hot fluhses | -87.500 | 87.500 | 21.0178 | 10.5089 |
| Nausea | -100.000 | 75.000 | 10.6878 | 5.3439 |
| Bladder control | -87.500 | 87.500 | 17.3068 | 8.6534 |
| Vaginal problems | -100.000 | 100.000 | 24.8593 | 12.4296 |
| Musculosceletal pain | -100.000 | 75.000 | 21.8886 | 10.9443 |
| Cognitive problems | -91.667 | 66.667 | 17.5353 | 8.7677 |
| Weight problems | -100.000 | 87.500 | 23.0196 | 11.5098 |
| Arm problems | -100.000 | 75.000 | 16.0042 | 8.0021 |
| **LASA QoL symptom indicators** |  |  |  |  |
| Tiredness | -99.000 | 100.000 | 26.7655 | 13.3827 |
| Sleep disturbances | -99.000 | 100.000 | 27.6927 | 13.8463 |
| Loss of sexual interest | -99.000 | 100.000 | 32.4739 | 16.2369 |
| Difficulties becoming aroused | -93.000 | 89.000 | 25.1513 | 12.5757 |
| **LASA QoL global indicators** |  |  |  |  |
| Physical well-being | -97.000 | 100.000 | 25.0709 | 12.5354 |
| Mood | -99.000 | 100.000 | 25.1533 | 12.5767 |
| Coping effort | -100.000 | 100.000 | 22.3820 | 11.1910 |
| Treatment burden | -100.000 | 100.000 | 25.8798 | 12.9399 |

**Note:** BCPT symptom scales were recalculated to 0-100 range before calculating the change.

**Table S3**: QoL completion and submission rates according to treatment and follow-up month, for the QoL analysis population

|  | Continuous letrozole (Arm A) | Intermittent letrozole (Arm B) | **Overall** |
| --- | --- | --- | --- |
| QL Submission Rate by Evaluation Month | 98.9 | 98.0 | 98.4 |
| 0 |  |  |  |
| 6 | 91.8 | 90.3 | 91.0 |
| 12 | 92.8 | 90.9 | 91.8 |
| 18 | 88.8 | 88.0 | 88.4 |
| 24 | 89.8 | 88.4 | 89.0 |
| **Overall Submission Rate** | 92.5 | 91.2 | 91.8 |
| **Number of Patients Completed All Expected QL Submissions** | 343 | 362 | 705 |

**Table S4**: Descriptive changes from baseline to month 12 and month 24 in symptom and global QoL scales

|  | **Continuous letrozole** | | | | **Intermittent letrozole** | | | |
| --- | --- | --- | --- | --- | --- | --- | --- | --- |
| **Change from baseline** | **Month 12** | | **Month 24** | | **Month 12** | | **Month 24** | |
| **BCPT Symptom Scales** | Mean | SD | Mean | SD | Mean | SD | Mean | SD |
| Hot flushes | 1.2 | 20.0 | 0.6 | 21.4 | 3.3 | 21.2 | 3.5 | 21.3 |
| Nausea | -0.8 | 10.8 | 0.3 | 10.1 | -0.5 | 10.3 | -0.6 | 10.7 |
| Bladder control | -1.5 | 17.2 | -2.2 | 18.9 | -1.6 | 15.7 | -1.9 | 17.4 |
| Vaginal problems | -7.5 | 26.6 | -6.3 | 26.5 | -3.6 | 22.2 | -4.7 | 25.2 |
| Musculoskeletal pain | -5.5 | 23.8 | -5.2 | 22.6 | -2.0 | 21.5 | -3.3 | 21.0 |
| Cognitive problems | -3.2 | 18.3 | -2.4 | 16.8 | -2.8 | 17.3 | -3.0 | 17.7 |
| Weight problems | 0.9 | 24.3 | 3.0 | 24.0 | 0.4 | 22.0 | -0.2 | 22.4 |
| Arm problems | 0.2 | 16.0 | 0.3 | 16.9 | 0.3 | 14.9 | 1.6 | 17.1 |
| **LASA QoL symptom indicators** |  |  |  |  |  |  |  |  |
| Hot flushes | 1.4 | 26.7 | 0.5 | 25.1 | 3.5 | 27.4 | 5.1 | 26.4 |
| Tiredness | -4.9 | 27.4 | -1.9 | 25.5 | -1.8 | 26.3 | -1.8 | 26.9 |
| Sleep disturbances | -4.8 | 29.5 | -4.5 | 27.2 | 0.5 | 25.8 | -1.8 | 28.3 |
| Loss of sexual interest | -3.6 | 31.7 | -4.0 | 34.4 | -0.3 | 30.6 | -1.7 | 33.6 |
| Difficulties becoming aroused | -6.5 | 27.0 | -10.5 | 26.2 | -3.9 | 24.1 | -7.9 | 25.8 |
| **LASA QoL global indicators** |  |  |  |  |  |  |  |  |
| Physical well-being | -6.1 | 25.9 | -5.4 | 25.4 | -1.9 | 22.8 | -3.0 | 22.9 |
| Mood | -5.8 | 26.0 | -4.3 | 24.3 | -1.8 | 25.0 | -2.5 | 24.8 |
| Coping effort | -0.6 | 21.7 | -0.2 | 22.3 | 0.1 | 23.1 | 2.9 | 22.2 |
| Treatment burden | -3.6 | 27.3 | -1.8 | 25.9 | 0.0 | 26.1 | 1.5 | 26.2 |

**Note:** BCPT symptom scales were recalculated to 0-100 range before calculating the change.

**Table S5** Differences between treatment groups in changes of symptom and global QoL scales from baseline

|  | **Estimated Difference between Treatment Groups (Intermittent minus Continuous)** | | | | | | | |
| --- | --- | --- | --- | --- | --- | --- | --- | --- |
|  | **12 months** | | | | **24 months** | | | |
|  | Mean | 95% LCL | 95% UCL | P-value | Mean | 95% LCL | 95% UCL | P-value |
| **BCPT Symptom Scales** |  |  |  |  |  |  |  |  |
| Hot flushes | 2 | -1 | 5 | 0.11 | 3 | 0 | 6 | 0.025 |
| Nausea | 0 | -1 | 2 | 0.63 | -1 | -2 | 1 | 0.29 |
| Bladder control | -0 | -2 | 2 | 0.97 | -0 | -3 | 2 | 0.92 |
| Vaginal problems | 4 | 1 | 8 | 0.017 | 2 | -2 | 5 | 0.33 |
| Musculoskeletal pain | 3 | 0 | 6 | 0.023 | 2 | -1 | 5 | 0.16 |
| Cognitive problems | 0 | -2 | 3 | 0.82 | -1 | -3 | 2 | 0.61 |
| Weight problems | -0 | -4 | 3 | 0.77 | -3 | -6 | 0 | 0.078 |
| Arm problems | -0 | -2 | 2 | 0.89 | 1 | -1 | 4 | 0.28 |
| **LASA symptom scales** |  |  |  |  |  |  |  |  |
| Hot flushes | 2 | -1 | 6 | 0.19 | 5 | 1 | 8 | 0.009 |
| Sleep disturbance | 5 | 1 | 9 | 0.0073 | 3 | -1 | 7 | 0.17 |
| Tiredness | 2 | -1 | 6 | 0.19 | -1 | -5 | 3 | 0.61 |
| Difficulties in becoming aroused | 5 | -1 | 11 | 0.11 | 4 | -2 | 10 | 0.20 |
| Loss of sexual interest | 4 | -1 | 8 | 0.097 | 2 | -3 | 8 | 0.34 |
| **LASA global scales** |  |  |  |  |  |  |  |  |
| Physical well-being | 4 | 1 | 8 | 0.0080 | 2 | -1 | 5 | 0.27 |
| Mood | 4 | 0 | 7 | 0.026 | 2 | -2 | 5 | 0.37 |
| Coping effort | 1 | -2 | 4 | 0.62 | 3 | -0 | 6 | 0.067 |
| Treatment burden | 3 | -0 | 7 | 0.082 | 3 | -0 | 7 | 0.082 |

Abbreviations: BCPT =Breast Cancer Prevention Trials; LASA =Linear Analogue Self-Assessment; LCL=lower confidence limit; UCL=upper confidence limit

All scales range from 0 to 100. BCPT symptom scales were recalculated to 0-100 range before calculating the change). Mean < 0 indicates continuous letrozole had greater worsening than intermittent letrozole. Mean differences between treatment groups, 95% CIs and Wald P-values were estimated with mixed-models contrasts for testing the differences versus zero at 12 months, and 24 months.

**Table S6.** Response analysis characterizing patients according to improved, stable or worsened quality-of-life scores

|  | **12 months** | | | | | | **24 months** | | | | | |
| --- | --- | --- | --- | --- | --- | --- | --- | --- | --- | --- | --- | --- |
|  | **Continuous letrozole** | | | **Intermittent letrozole** | | | **Continuous letrozole** | | | **Intermittent letrozole** | | |
|  | **%** | **95% LCL** | **95% UCL** | **%** | **95% LCL** | **95% UCL** | **%** | **95% LCL** | **95% UCL** | **%** | **95% LCL** | **95% UCL** |
| **BCPT**  **hot flushes** |  |  |  |  |  |  |  |  |  |  |  |  |
| Worsened | 25.7% | 21.6% | 29.7% | 20.6% | 17.1% | 24.2% | 27.0% | 22.8% | 31.2% | 22.8% | 19.0% | 26.6% |
| Stable | 33.9% | 29.5% | 38.3% | 36.6% | 32.4% | 40.9% | 29.1% | 24.8% | 33.3% | 28.1% | 24.0% | 32.1% |
| Improved | 31.7% | 27.4% | 36.0% | 29.8% | 25.7% | 33.8% | 30.9% | 26.5% | 35.2% | 34.0% | 29.7% | 38.2% |
| Unknown | 8.7% | 6.1% | 11.3% | 13.0% | 10.0% | 15.9% | 13.0% | 9.9% | 16.2% | 15.2% | 11.9% | 18.4% |
| **BCPT**  **vaginal problems** |  |  |  |  |  |  |  |  |  |  |  |  |
| Worsened | 34.2% | 29.7% | 38.6% | 29.8% | 25.7% | 33.8% | 34.8% | 30.3% | 39.3% | 28.7% | 24.6% | 32.8% |
| Stable | 34.2% | 29.7% | 38.6% | 32.6% | 28.4% | 36.7% | 28.8% | 24.6% | 33.1% | 31.9% | 27.6% | 36.1% |
| Improved | 18.3% | 14.7% | 21.9% | 18.8% | 15.4% | 22.3% | 17.8% | 14.2% | 21.5% | 19.4% | 15.8% | 23.0% |
| Unknown | 13.4% | 10.2% | 16.6% | 18.8% | 15.4% | 22.3% | 18.5% | 14.9% | 22.2% | 20.0% | 16.4% | 23.7% |
| **BCPT musculoskeletal pain** |  |  |  |  |  |  |  |  |  |  |  |  |
| Worsened | 30.8% | 26.5% | 35.1% | 26.7% | 22.8% | 30.6% | 27.5% | 23.3% | 31.7% | 26.4% | 22.4% | 30.4% |
| Stable | 43.1% | 38.5% | 47.7% | 39.3% | 34.9% | 43.6% | 44.2% | 39.5% | 48.8% | 42.6% | 38.1% | 47.1% |
| Improved | 17.4% | 13.9% | 20.9% | 21.3% | 17.6% | 24.9% | 15.3% | 11.9% | 18.7% | 16.0% | 12.7% | 19.3% |
| Unknown | 8.7% | 6.1% | 11.3% | 12.8% | 9.8% | 15.7% | 13.0% | 9.9% | 16.2% | 15.0% | 11.8% | 18.2% |
| **LASA**  **sleep disturbance** |  |  |  |  |  |  |  |  |  |  |  |  |
| Worsened | 26.3% | 22.2% | 30.4% | 18.6% | 15.2% | 22.1% | 26.5% | 22.4% | 30.7% | 22.6% | 18.8% | 26.4% |
| Stable | 44.9% | 40.2% | 49.5% | 48.4% | 44.0% | 52.8% | 40.7% | 36.1% | 45.4% | 42.6% | 38.1% | 47.1% |
| Improved | 16.5% | 13.1% | 20.0% | 19.2% | 15.7% | 22.7% | 17.4% | 13.8% | 21.0% | 20.5% | 16.8% | 24.1% |
| Unknown | 12.3% | 9.2% | 15.3% | 13.8% | 10.7% | 16.8% | 15.3% | 11.9% | 18.7% | 14.3% | 11.2% | 17.5% |
| **LASA**  **physical-wellbeing** |  |  |  |  |  |  |  |  |  |  |  |  |
| Worsened | 29.2% | 25.0% | 33.5% | 22.3% | 18.6% | 25.9% | 28.1% | 23.9% | 32.4% | 23.2% | 19.4% | 27.0% |
| Stable | 46.4% | 41.8% | 51.1% | 49.2% | 44.8% | 53.6% | 42.6% | 37.9% | 47.2% | 47.0% | 42.5% | 51.6% |
| Improved | 15.4% | 12.0% | 18.8% | 16.4% | 13.1% | 19.7% | 15.8% | 12.4% | 19.2% | 16.0% | 12.7% | 19.3% |
| Unknown | 8.9% | 6.3% | 11.6% | 12.1% | 9.3% | 15.0% | 13.5% | 10.3% | 16.7% | 13.7% | 10.6% | 16.8% |
| **LASA**  **mood** |  |  |  |  |  |  |  |  |  |  |  |  |
| Worsened | 26.8% | 22.7% | 30.9% | 21.1% | 17.4% | 24.7% | 25.2% | 21.1% | 29.3% | 23.8% | 20.0% | 27.7% |
| Stable | 49.8% | 45.1% | 54.4% | 49.8% | 45.4% | 54.2% | 47.6% | 42.9% | 52.3% | 44.1% | 39.6% | 48.6% |
| Improved | 13.4% | 10.2% | 16.6% | 16.2% | 12.9% | 19.5% | 12.4% | 9.3% | 15.5% | 18.1% | 14.7% | 21.6% |
| Unknown | 10.0% | 7.3% | 12.8% | 13.0% | 10.0% | 15.9% | 14.9% | 11.5% | 18.2% | 13.9% | 10.8% | 17.1% |

BCPT=Breast Cancer Prevention Trial; LASA=linear analogue self-assessment; LCL=lower confidence limit; UCL=upper confidence limit

**Section 3: Exploratory analysis to address association between the BCPT hot flushes scale and the corresponding LASA indicator.**

In an exploratory analysis we addressed the association between the BCPT hot flushes scale and the corresponding LASA indicator.

**Table S7**.Correlations between LASA indicator for hot flushes and BCPT hot flushes scale and single items

|  | **LASA hot flushes** | | |
| --- | --- | --- | --- |
| **Baseline** | **N** | **Pearson Correlation Coefficients** | **p** |
| BCPT hot flushes scale | 932 | 0.82 | <.0001 |
| BCPT single-item hot flushes | 924 | -0.85 | <.0001 |
| BCPT single-item night sweats | 927 | -0.65 | <.0001 |
| **Month 24** |  |  |  |
| BCPT hot flushes scale | 777 | 0.81 | <.0001 |
| BCPT single-item hot flushes | 772 | -0.84 | <.0001 |
| BCPT single-item night sweats | 769 | -0.63 | <.0001 |

**Note:** Correlation coefficients are considered low (<0.4) moderate (0.4 – 0.7) and high (>0.7). BCPT symptom scales were recalculated to 0-100 range before calculating the correlations).

BCPT=Breast Cancer Prevention Trial; LASA=linear analogue self-assessment.

**Figure S1**. Changes in LASA hot flushes and BCPT hot flushes (scale was recalculated to 0-100 range) for the two treatment groups. Effect sizes were calculated as changes from baseline (month 24minus baseline) for each of the measures and each treatment group. For all measures, positive effect sizes indicate an increase in scores (improvement of condition).
